# Altered sensorimotor-occipital network topology in chronic insomnia: A graph-theoretical and edge-wise resting-state fMRI analysis

**DOI:** 10.1016/j.nicl.2026.104084

**Published:** 2026-10-31

**Authors:** Zuguang Wen, Yuefeng Kong, Yaxuan Wen, Hui Zhang, Liling Long

**Affiliations:** a First Affiliated Hospital of Guangxi Medical University, No. 6 Shuang-yong Road, Nanning, Guangxi 530021, China; b Liaoning University of Traditional Chinese Medicine, Shenyang, Liaoning, China; c MR Research Collaboration Team, Siemens Healthineers Ltd., Shenzhen, China

**Keywords:** Chronic insomnia, Graph-theoretical analysis, Edge-wise analysis, Sensorimotor network, Posterior occipital cortex

## Abstract

**Objective:**

The multi-level network aberrations in chronic insomnia (CI) and their neurobiological underpinnings remain elusive. Here, we applied graph-theoretical, edge-wise, and modular analyses to resting-state fMRI data to characterize systems-level functional network reorganization in CI patients.

**Methods:**

Resting-state fMRI was acquired from CI patients and healthy controls (HCs). Whole-brain functional networks were parcellated using the Dosenbach-160 atlas, and then graph-theoretical, edge-wise, and modular analyses were performed. Group differences in global, nodal, edge-wise, and modular metrics were examined via a general linear model, with age, sex, education, and framewise displacement as covariates. Global and nodal metrics were corrected using the Bonferroni method; edge-wise comparisons used network-based statistics (NBS; cluster-forming threshold *p* < 0.001, 5000 permutations, FWE-corrected *p* < 0.05).

**Results:**

Compared with HCs, CI patients showed significantly decreased average clustering coefficients, shorter path lengths, and reduced local efficiency, but increased global efficiency – a pattern indicative of a shift toward random network topology. Notably, normalized small-worldness scalar remained intact. Nodal clustering decreased in the left posterior occipital cortex. Edge-wise analysis revealed widespread connectivity disruptions involving parietal–posterior occipital, posterior insular–occipital, and dorsolateral prefrontal–temporal pathways. Modular analyses further demonstrated reduced connectivity both between sensorimotor and occipital networks, and within the sensorimotor network itself.

**Conclusions:**

CI entails multi-level functional network reorganization, characterized by global topological randomization, reduced occipital nodal clustering, and selective decoupling within sensorimotor and sensorimotor–occipital circuits. These findings suggest that CI involves widespread integration failure across large-scale systems rather than isolated regional dysfunction, with potential implications for understanding its neurocognitive sequelae.

## Highlights

• Multi-level graph analysis reveals topological reorganization in insomnia, with reduced local but increased global efficiency.
• Nodal clustering is reduced in the left posterior occipital cortex, implicating local hub dysfunction.
• Edge-wise and modular analyses revealed decoupling between sensorimotor and occipital networks.
• Chronic insomnia shows widespread functional disturbance from circuit-level coupling failure, beyond limbic models.
• Network metrics serve as neuroimaging biomarkers and targets for network neuromodulation.

## Graphical abstract

**Figure:**
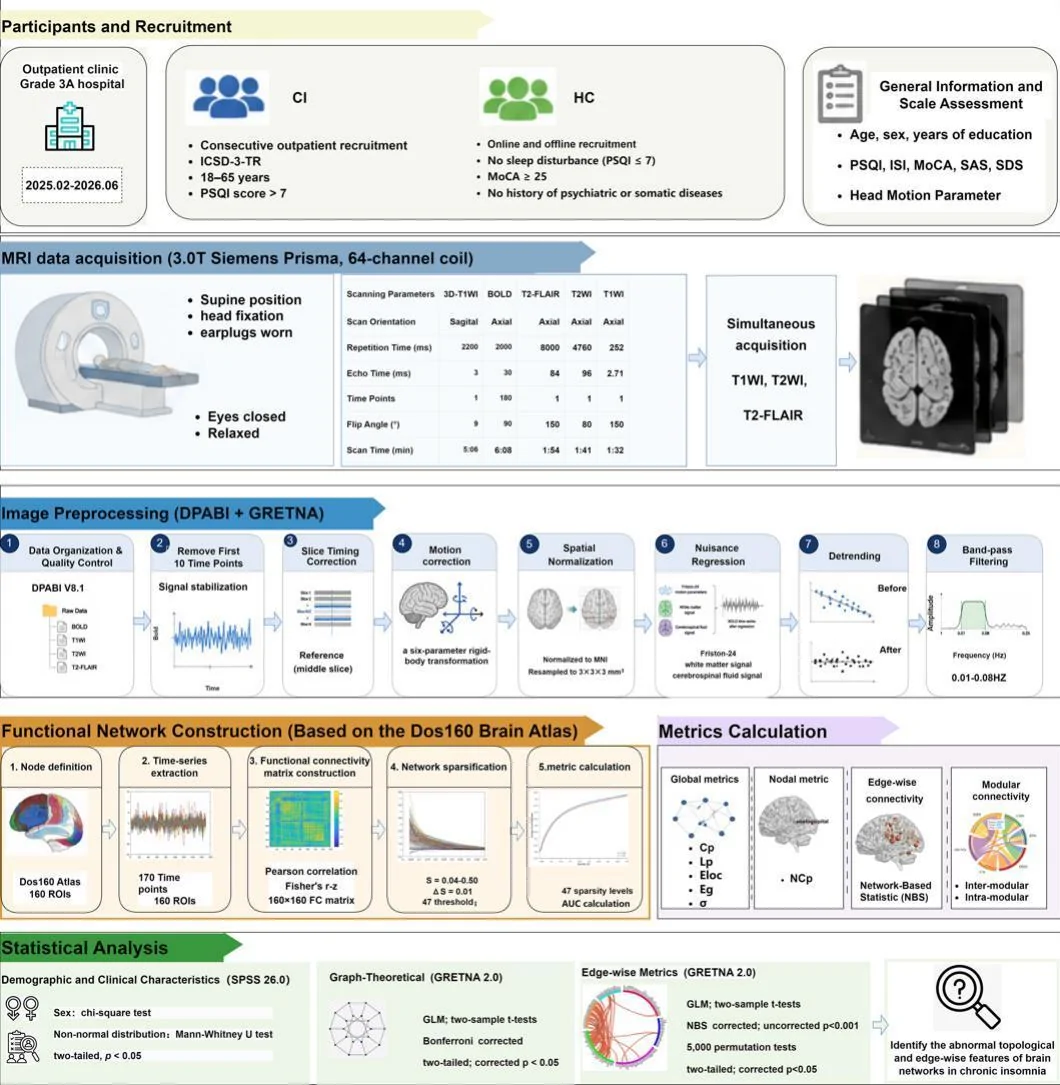

## List of abbreviations

**Table:** 

3D-T1WI	Three-dimensional T1-weighted Imaging
AUC	Area Under the Curve
BOLD	Blood Oxygenation Level-Dependent
CI	Chronic Insomnia
C_P_	Average Clustering Coefficient
E_g_	Global Efficiency
E_loc_	Average Local Efficiency
FD	Framewise Displacement
FWE	Family-Wise Error Rate
GLM	General Linear Model
HC	Healthy Controls
ICSD	International Classification of Sleep Disorders
ISI	Insomnia Severity Index
L_P_	Average Shortest Path Length
MNI	Montreal Neurological Institute
MoCA	Montreal Cognitive Assessment
MRI	Magnetic Resonance Imaging
NBS	Network-Based Statistic
N_cp_	Nodal Clustering Coefficient
ON	Occipital Net
PSQI	Pittsburgh Sleep Quality Index
ROIs	Regions Of Interest
SAS	Self-Rating Anxiety Scale
SDS	Self-Rating Depression Scale
SMN	Sensorimotor Net
T1WI	T1-Weighted Imaging
T2-FLAIR	T2-Fluid-Attenuated Inversion Recovery
T2WI	T2-Weighted Imaging
γ	Normalized Clustering Coefficient
λ	Normalized Path Length
σ	Small-World Coefficient

Chronic insomnia (CI) is one of the most prevalent sleep disorders, with a global prevalence of approximately 16.2% among adults (Benjafield et al., 2025), and it poses a significant public health burden. Persistent sleep disturbance not only impairs emotional, quality-of-life domains, and daytime cognitive but is also associated with increased risks of endocrine-metabolic disturbances and psychiatric disorders such as depression and anxiety (Weihs et al., 2023). Nevertheless, the neurobiological mechanisms underlying the onset and progression of CI remain poorly understood (Gong et al., 2023; Zheng et al., 2023).

With advances in neuroimaging techniques, accumulating evidence has revealed CI-related brain alterations at multiple levels, including structural changes, regional spontaneous activity abnormalities, and disrupted functional connectivity (Wang et al., 2025a; Zhang et al., 2022). These findings have contributed to prevailing models that emphasize hyperarousal and sleep–wake circuitry dysfunction as core mechanisms of CI (Riemann et al., 2010; Saper et al., 2005; Yu et al., 2025). However, most studies have focused on isolated regions or specific connections, leaving whole-brain network organization and cross–network interactions poorly understood (Liu et al., 2018). Graph-theoretical studies have yielded inconsistent findings, largely due to their predominant focus on the default–mode, frontal–limbic, and attention networks (Yin et al., 2026), with insufficient attention to sensorimotor and occipital networks. Moreover, most analyses rely on global topological metrics (Lin et al., 2023a), rarely integrating nodal, edge–wise, or modular features, thereby limiting a comprehensive characterization of network reorganization. Consequently, whether CI involves persistent topological remodeling, hub dysfunction, or inter–module communication breakdown remains largely unexplored.

Graph-theoretical and edge-wise network analyses provide powerful frameworks for characterizing brain network architecture across multiple levels, including global topological, nodal, edge-wise, and modular analyses (Mamat et al., 2024). Therefore, the present study integrated these complementary approaches to systematically characterize whole-brain functional network alterations in patients with CI. By adopting a multi-scale network perspective, we aimed to elucidate the large-scale neural mechanisms underlying CI and identify potential neuroimaging biomarkers, with particular attention to sensorimotor and occipital systems given their underrepresentation in prior work.

## 1 Materials and methods

### 1.1 Participants and clinical assessment

This prospective case-control study consecutively enrolled patients with a clinical diagnosis of chronic insomnia at the outpatient clinic of the First Affiliated Hospital of Guangxi Medical University between February 2025 and June 2026. Healthy controls (HCs) who did not differ significantly from patients in age or sex were recruited through both online and in-person recruitment. The study design was illustrated in Fig. 1.

**Fig. 1:**
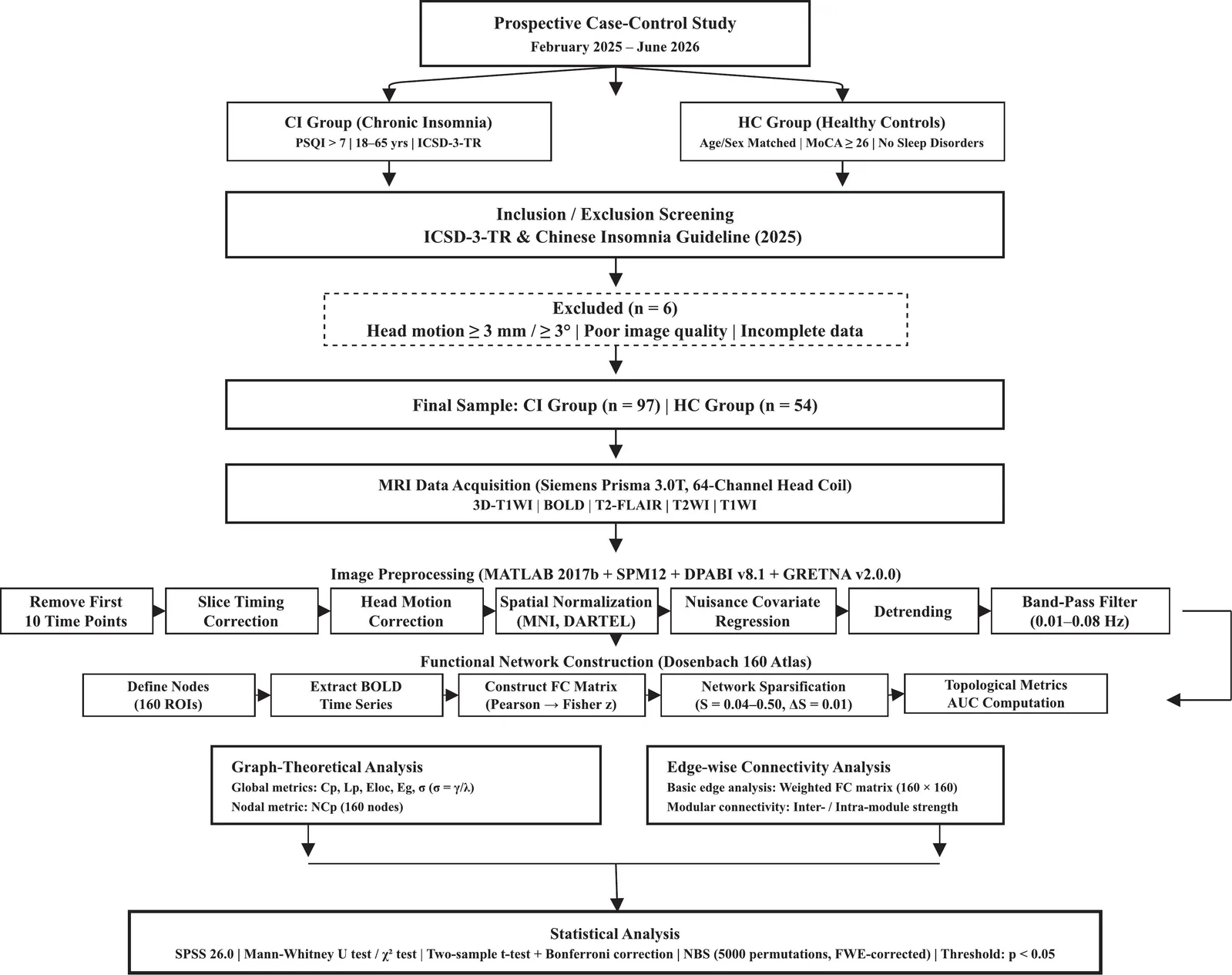
Flowchart illustrating the study design and participant selection process.

All participants were right-handed, as assessed by the Edinburgh Handedness Inventory (Oldfield, 1971). They were instructed to abstain from alcohol, caffeine, and other stimulants for at least one week before scanning. Any acute illness or vigorous exercise within the preceding week was documented. Prior to the experiment, all participants received a detailed explanation of the study purpose and MRI safety screening procedures, and written informed consent was obtained from each individual. The study was conducted in accordance with the Declaration of Helsinki and approved by the Ethics Committee of the First Affiliated Hospital of Guangxi Medical University (Approval No. 2025-K0070).

Demographic and clinical data collected for each participant comprised (1) demographic characteristics, including age, sex, and years of education; (2) neuropsychological assessments, including the Pittsburgh Sleep Quality Index (PSQI), Insomnia Severity Index (ISI), Montreal Cognitive Assessment (MoCA), Self-Rating Anxiety Scale (SAS), and Self-Rating Depression Scale (SDS); (3) imaging quality metrics, including mean framewise displacement (FD) calculated during image preprocessing.

### 1.2 Diagnostic criteria, inclusion criteria, and exclusion criteria

The diagnosis of chronic insomnia was based strictly on the I*nternational Classification of Sleep Disorders, Third Edition, Text Revision* (ICSD-3-TR) (Patel, 2023; Sateia, 2014) and the *Chinese Guideline for the Diagnosis and Treatment of Insomnia* (2025 edition) (Zhang, 2025). All participants were independently evaluated by two neurologists with over 10 years of clinical experience through semi-structured interviews covering sleep and mental status, with final diagnoses confirmed by integrating clinical history and scores from the PSQI.

**Inclusion criteria for the CI group:**

1. Predominant sleep complaints including difficulty initiating or maintaining sleep, or early-morning awakening, accompanied by daytime functional impairments such as fatigue, reduced attention, or emotional dysregulation.
2. Insomnia symptoms occurring at least three times per week for a duration of ≥3 months.
3. Age between 18 and 65 years.
4. PSQI score > 7.
5. Able to complete neuropsychological assessments and MRI scanning.

**Inclusion criteria for the HC group:**

1. Participants were not matched at the design stage，but did not differ significantly in age or sex.
2. No sleep disturbances confirmed by PSQI score, sleep habit questionnaires, and clinical interviews.
3. No personal or family history of psychiatric disorders (SAS < 50, SDS < 53).
4. No severe chronic physical illnesses, history of substance abuse, or conditions affecting central nervous system function.
5. MoCA score ≥ 26, indicating normal cognitive function.

**Exclusion criteria and Post-hoc Exclusion Criteria:**

Participants were excluded if they met any of the following:

1. Other sleep disorders with identifiable etiologies or triggers.
2. Use of sedative-hypnotics or other medications affecting central nervous system function within the past month.
3. Past or current psychiatric disorders, substance dependence, or related history.
4. Central nervous system diseases or severe cardiac, hepatic, renal, or other major organ disorders.
5. Receipt of treatments that might affect brain function (e.g., acupuncture or rTMS) within the past month, or participation in other clinical trials within the past 2 weeks.

Additional exclusions were applied for: 1. falling asleep, excessive head motion, or inability to maintain a resting state during MRI scanning; 2. intracranial organic lesions detected on conventional MRI; 3. incomplete clinical data or neuropsychological assessments; 4. head motion exceeding preset thresholds (translation ≥3 mm, or rotation ≥3° (Yin et al., 2026)) or unsatisfactory image quality.

After applying the inclusion/exclusion criteria and quality control, six participants were excluded: three due to incomplete clinical data (HC: *n* = 2; CI: *n* = 1), one due to intracranial structural abnormalities (CI), one due to poor compliance (CI), and one due to unsatisfactory image quality (CI). The final sample comprised 97 patients with CI and 54 healthy controls.

### 1.3 MRI data acquisition

All MRI data were acquired using a Siemens Prisma 3.0 T scanner equipped with a 64-channel head coil. Participants were positioned supine with their heads secured by straps and foam pads, and wore earplugs to reduce scanner noise. Before acquisition, participants were instructed to remain relaxed with eyes closed, stay awake, and minimize movement. If a participant developed tachycardia, agitation, or any condition that prevented them from tolerating the scan, the scan was immediately discontinued. Additionally, T1-weighted imaging (T1WI), T2-weighted imaging (T2WI), and T2-weighted fluid-attenuated inversion recovery (T2-FLAIR) sequences were acquired to exclude intracranial organic lesions. Detailed acquisition parameters were provided in Table 1.

**Table 1:** MRI scanning parameters.

Scanning parameters	3D-T1WI	BOLD	T2-FLAIR	T2WI	T1WI
Scan orientation	Sagital	Axial	Axial	Axial	Axial
Repetition time (ms)	2200	2000	8000	4760	252
Echo time (ms)	3	30	84	96	2.71
Field of view (mm)	256 × 256	220 × 220	220 × 185.6	220 × 185.6	220 × 192.5
Slice thickness (mm)	1	4	5	5	5
Slice gap (mm)	no gap	no gap	1	1	1
Slice number	176	36	24	24	24
Voxel size (mm)	1 × 1 × 1	3 × 3 × 4	0.7 × 0.7 × 5	0.2 × 0.2 × 5	0.4 × 0.4 × 5
Matrix	256 × 256	74 × 74	189 × 320	265 × 448	224 × 256
Time points	1	180	1	1	1
Flip angle (°)	9	90	150	80	150
Scan time (min)	5:06	6:08	1:54	1:41	1:32

3D-T1WI: Three-dimensional T1-weighted Imaging; BOLD: Blood oxygenation level-dependent; T2-FLAIR: T2-weighted fluid-attenuated inversion recovery imaging; T2WI: T2-weighted imaging; T1WI: T1-weighted imaging.

### 1.4 Image preprocessing

Image preprocessing was performed using MATLAB (R2017b, The MathWorks, Natick, MA, USA) and Statistical Parametric Mapping version 12 (SPM12). Data organization, format conversion, and quality control were conducted with DPABI v8.1 (Yan et al., 2016), after which preprocessing was performed in GRETNA v2.0.0 (Wang et al., 2015) according to the following procedures: (1) removal of the first 10 time points to allow signal stabilization; (2) slice timing correction with interleaved acquisition, using the middle slice as reference; (3) motion correction using a six-parameter rigid-body transformation, and participants with head translation ≥3 mm or rotation ≥3° excluded; (4) spatial normalization to the Montreal Neurological Institute (MNI) standard space using the DARTEL two-step registration; (5) regression of nuisance covariates including Friston-24 motion parameters, white matter signal, and cerebrospinal fluid signal, because global signal regression (GSR) remains controversial in resting-state functional connectivity analyses and may systematically affect between-group comparisons of graph-theoretical metrics, GSR was not performed in this study; (6) detrending to remove low-frequency drifts; and (7) band-pass filtering (0.01–0.08 Hz).

## 2 Functional network construction and topological metric derivation

### 2.1 Node definition

Nodes were defined using the Dosenbach-160 functional brain atlas (Dosenbach et al., 2010), which divides the whole brain into 160 regions of interest (ROIs) encompassing cortical, subcortical, and cerebellar areas. We selected the Dosenbach-160 atlas for the following reasons: First, it is a functional parcellation based on both resting-state and task-based fMRI data, ensuring high functional homogeneity within nodes. Second, it has been widely used in developmental, clinical, and resting-state network studies, facilitating comparison with previous findings. Third, its resolution (160 regions) strikes a balance between spatial specificity and computational feasibility. Fourth, its functional network parcellation (e.g., SMN, ON) aligns closely with the networks of interest in the present study.

### 2.2 Time-series extraction

For each participant, the mean BOLD time series was extracted by averaging signals across all voxels within each ROI and used as the nodal representative time series, yielding a 160 × 170 (ROI × time points) matrix.

### 2.3 Functional connectivity matrix construction

Pairwise Pearson correlation coefficients (*r*) between all ROI time series were computed to construct a 160 × 160 (ROI × ROI) symmetric functional connectivity matrix. Correlation coefficients were then converted to *z*-scores using Fisher's *r*-to-*z* transformation to improve normality and stabilize variance:

$$z=0.5\ln\left(\frac{1+r}{1-r}\right)$$

Diagonal elements (self-connections) were excluded from subsequent analyses.

### 2.4 Network sparsification

To minimize inter-individual variability in connection density, binary networks were thresholded using a sparsity (*S*) approach, where *S* represents the proportion of retained edges out of all possible edges. The sparsity range was determined using GRETNA's built-in functions and validated by one-sample *t*-tests and network connectivity criteria, following previous studies (Dosenbach et al., 2010; Wang et al., 2015). A range of *S* = 0.04–0.50 with an increment of *ΔS* = 0.01 yielded 47 threshold levels. At each level, the top *S*-fraction of strongest connections were retained (set to 1) and the rest were set to 0, generating binary undirected networks for each participant.

### 2.5 Topological metric calculation and summarization

To reduce threshold-dependent bias, network metrics were computed at each of the 47 sparsity levels, and the area under the curve (AUC) for each metric was calculated as an integrated metric that reflects overall topological characteristics across the entire sparsity range. AUC-based metrics reduce dependence on threshold selection and enhance the robustness of between-group comparisons.

## 3 Graph-theoretical analysis

### 3.1 Global network metrics

To characterize the overall topological organization of whole-brain functional networks, the following global metrics were computed:

Average clustering coefficient (C_p_): reflects local clustering and information processing capacity; defined as the mean clustering coefficient across all nodes, where the clustering coefficient of a given node is the ratio of existing connections among its neighbors to the maximum possible number of connections (Lin et al., 2023b; Ma et al., 2023).

Average shortest path length (L_p_): defined as the mean of the shortest path lengths between all pairs of nodes, serving as a measure of global information transfer efficiency (Chen et al., 2024; Wang et al., 2020).

Average local efficiency (E_loc_): quantifying the information transfer capacity and fault tolerance within local subnetworks, defined as the mean of global efficiency computed over the neighborhood subnetwork of each node (Latora and Marchiori, 2001; Nasiri et al., 2025).

Global efficiency (E_g_): defined as the mean of the inverse of the shortest path lengths between all node pairs, evaluating the overall information transfer capacity of the network, and inversely related to Lₚ (Bullmore and Sporns, 2009; Latora and Marchiori, 2001).

Small-world properties: assessed by comparing the real network with matched random networks. The normalized clustering coefficient (*γ*) was defined as *γ* = C_p_/C_p_rand_, and the normalized path length (*λ*) as *λ* = L_p_/L_p_rand_, where C_p__rand and L_p__rand were averaged over 100 matched random networks. The small-world coefficient *σ* was computed as *σ* = *γ/λ*. A network was considered to exhibit small-world architecture if *γ* > 1, *λ* ≈ 1, and *σ* > 1 (Humphries and Gurney, 2008; Watts and Strogatz, 1998), thereby indicating a favorable balance between high local clustering and global efficiency at a relatively low wiring cost.

### 3.2 Nodal network metrics

To further characterize region-specific topological organization, the nodal clustering coefficient (NCp) was computed for each node. NCp was defined as the ratio of actual connections among a node's neighbors to the maximum possible number of connections, reflecting the degree of local clustering and information integration capacity of that node (Chen et al., 2024). A higher NCp indicates stronger functional coupling with neighboring nodes, suggesting a more prominent role in local network integration.

## 4 Edge-wise connectivity analysis

### 4.1 Basic edge-wise analysis

Basic edge-wise analysis was performed directly on the fully weighted functional connectivity matrices (160 × 160) after Fisher's *r*-to-*z* transformation, without thresholding or sparsity-based filtering, to identify between-group differences in edge-wise functional connectivity strength between specific ROI pairs.

### 4.2 Modular connectivity analysis

Modular connectivity analysis was conducted to characterize system-level connectivity aberrations across large-scale functional networks (Fasiello et al., 2022). Brain regions were assigned to functional modules based on the Dosenbach 160 atlas parcellation and the modular partitioning algorithm implemented in GRETNA. The following metrics were then calculated:

**Inter-modular connectivity strength**: defined as the mean Fisher z-value across all node pairs belonging to different modules, reflecting the level of information exchange and integration between distinct functional systems.

**Intra-modular connectivity strength**: defined as the mean Fisher z-value across all node pairs within the same module, assessing the functional synchrony and integration among nodes within each module.

### 4.3 Statistical analysis

#### 4.3.1 Demographic and clinical data

Statistical analyses were performed using IBM SPSS Statistics 26.0. Sex differences were assessed using the chi-square test, with results presented as frequencies (percentages) [n (%)]. Continuous variables, all of which deviated from normal distribution as confirmed by the Shapiro-Wilk test, were compared between groups using the Mann-Whitney *U* test and reported as medians (interquartile ranges) [M (P25, P75)]. All tests were two-tailed, with statistical significance set at *p* < 0.05.

#### 4.3.2 Statistical comparisons for graph-theoretical and edge-wise metrics

For global and nodal metrics, the AUC across 47 sparsity levels was used as the summary statistic, and intergroup differences were assessed using two-sample *t*-tests within a general linear model (GLM) framework with age, sex, years of education, and mean FD as covariates. Multiple comparisons were corrected using the Bonferroni method: the significance threshold was set at α = 0.05/5 = 0.01 for the five global metrics, and at α = 0.05/160 ≈ 0.000313 for nodal metrics.

For edge-wise connectivity, two-sample *t*-tests within the GLM framework were performed on the Fisher z-transformed functional connectivity matrices to compare intergroup differences in each edge, and multiple comparisons were controlled using the network-based statistic (NBS) approach (Zalesky et al., 2010). Specifically, an initial edge-level uncorrected threshold of *p* < 0.001 was applied to identify significant edges and form connected components, followed by 5000 permutation tests to control the family-wise error rate (FWE) at the component level.

For modular connectivity analyses, a total of 15 comparisons were performed for inter-modular connectivity and 6 comparisons for intra-modular connectivity. Bonferroni correction was applied, yielding corrected significance thresholds of *p* < 3.33 × 10^−3^ and *p* < 8.33 × 10^−3^, respectively.

All analyses were conducted using GRETNA. Details of multiple comparison correction are described above.

## 5 Results

### 5.1 Demographic and clinical characteristics

No significant differences were observed between the CI and HC groups in age, sex distribution, or FD (all *p* > 0.05). In contrast, significant between-group differences were found in years of education, and in PSQI, ISI, MoCA, SAS, and SDS scores (all *p* < 0.05). Detailed results and disease course were presented in Table 2 and Fig. 2.

**Table 2:** Demographic and clinical characteristics of HC and CI.

Characteristics	HC (54)	CI(97)	*p***value**	Statistics
Demographics				
Sex (F/M) ^(1)^	28(51.9%)/26(48.1%)	62(63.9%)/35(36.1%)	*p* **= 0.148**	2.097
Age (year) ^(2)^	31.00(28.00–38.25)	35.00(28.00–43.50)	*p* **= 0.300**	−1.036
Education (year) ^(2)^	20.00(16.00–21.00)	17.00(15.00–20.00)	*p* **= 0.027**	−2.216
FD ^(2)^	0.06(0.04–0.07)	0.06(0.04–0.07)	*p* **= 0.476**	−0.712
Duration ^(2)^	–	36.00(12.00–78.00)	*–*	–

Clinical **characteristics**				
PSQI ^(2)^	5.00(3.00–6.00)	12.00(10.00–14.00)	*p* **< 0.001**	−10.194
ISI ^(2)^	2.00(0.00–6.25)	12.00(9.00–15.00)	*p* **< 0.001**	−9.388
MoCA ^(2)^	29.00(28.00–29.00)	27.00(25.00–29.00)	*p* **< 0.001**	−3.701
SAS ^(2)^	31.00(26.00–36.00)	45.00(37.50–51.00)	*p* **< 0.001**	−7.418
SDS ^(2)^	45.50(37.00–50.00)	52.00(47.00–57.00)	*p* **< 0.001**	−6.052

HC: healthy control; CI: chronic insomnia; FD: framewise displacement; PSQI: Pittsburgh Sleep Quality Index; ISI: Insomnia Severity Index; MoCA: Montreal Cognitive Assessment; SAS: Self-Rating Anxiety Scale; SDS: Self-Rating Depression Scale. Statistical comparisons: (1) Chi-square test; (2) Mann-Whitney *U* test.

**Fig. 2:**
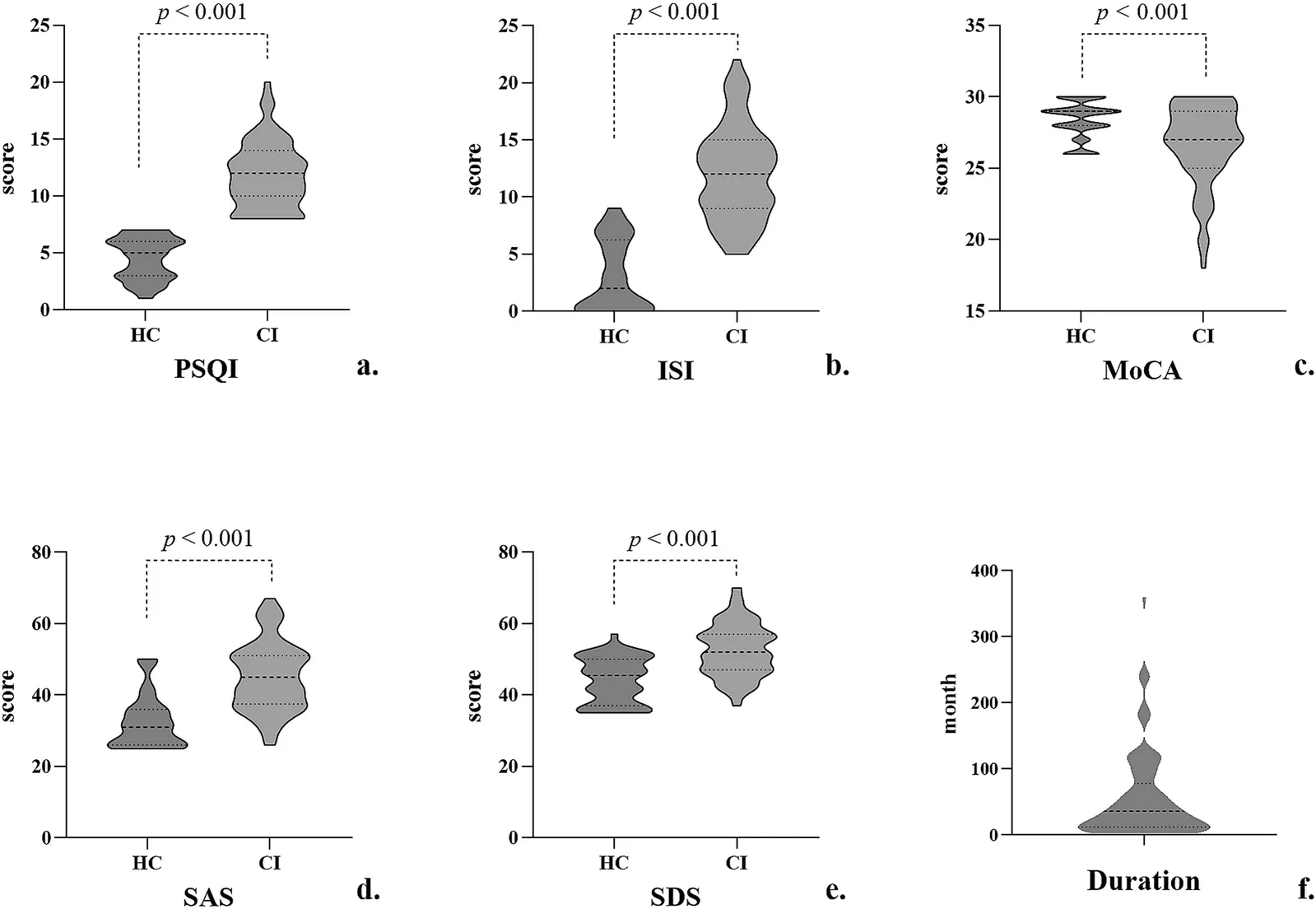
Group comparisons of clinical scale scores between HC and CI. Scales assessed include: PSQI: Pittsburgh Sleep Quality Index; ISI: Insomnia Severity Index; MoCA: Montreal Cognitive Assessment; SAS: Self-Rating Anxiety Scale; SDS: Self-Rating Depression Scale; HC: healthy control; CI: chronic insomnia.

## 6 Graph-theoretical results

The distribution curves of global network metrics across sparsity thresholds for both groups are shown in Fig. 3-1, Fig. 3-2. In terms of global topological properties, the chronic insomnia group exhibited significantly decreased average clustering coefficient, average shortest path length, and local efficiency, along with significantly increased global efficiency, relative to the healthy control group (all corrected *p* < 0.01). No significant between-group difference was observed in small-worldness (corrected *p* > 0.01; Fig. 3-3). At the nodal level, the chronic insomnia group showed a significantly reduced NCp in the left posterior occipital cortex compared with controls (corrected *p* < 3.13 × 10^−4^; Fig. 4). See Table 3 for details.

**Fig. 3-1:**
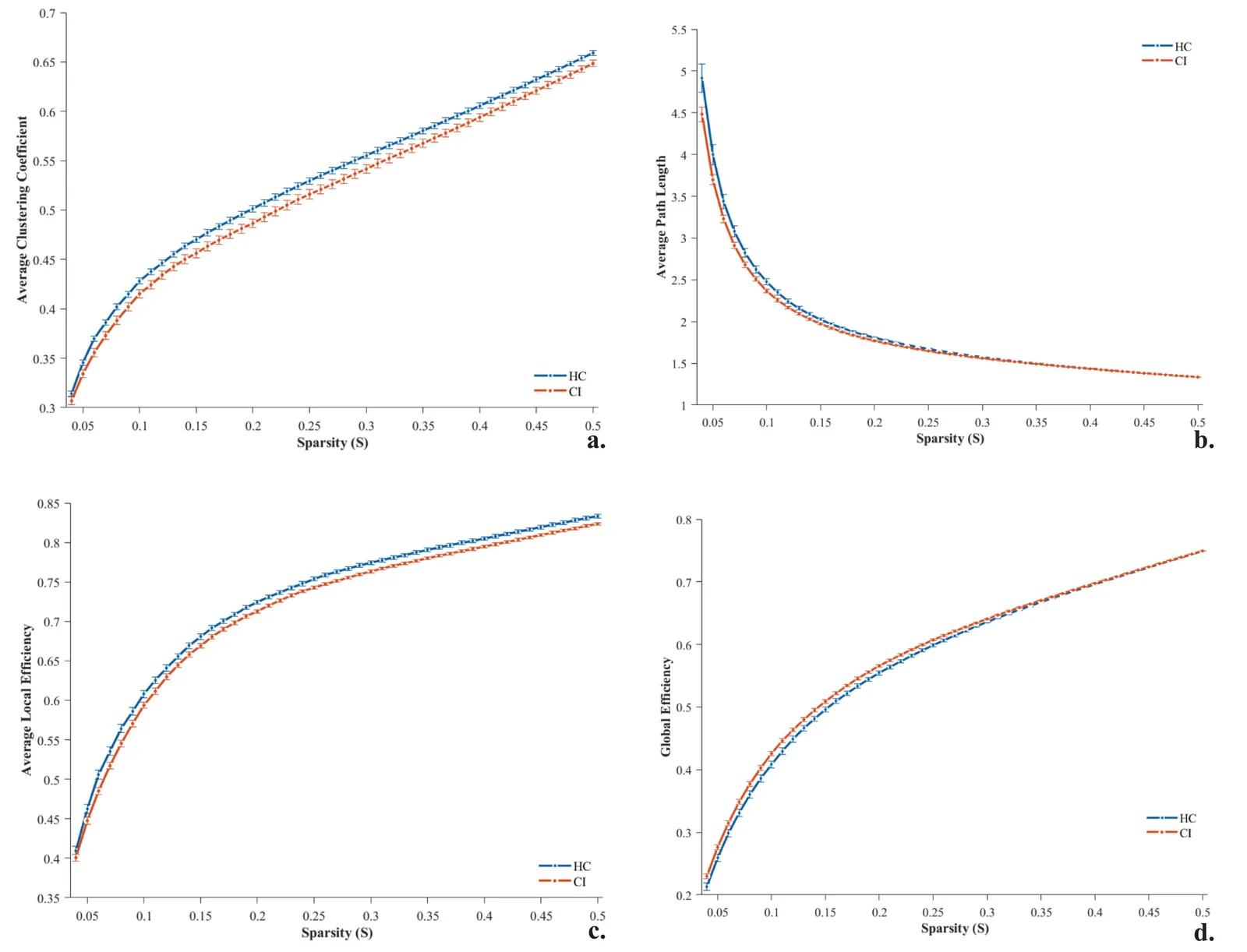
Global network metrics as a function of sparsity thresholds in HC and CI. Panels show: (a) average clustering coefficient, (b) average path length, (c) average local efficiency, and (d) global efficiency；HC: healthy control; CI: chronic insomnia.

**Fig. 3-2:**
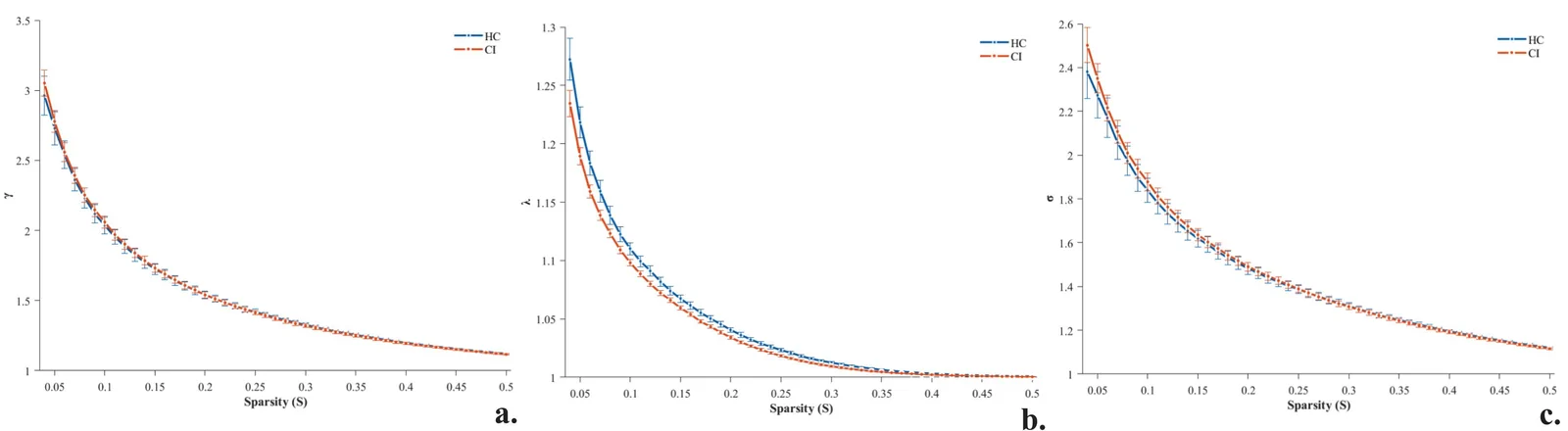
Small-world properties as a function of sparsity thresholds in HC and CI. Panels show: (a) γ, the normalized clustering coefficient; (b) λ, the normalized characteristic path length; and (c) σ, the small-worldness coefficient (calculated as γ/λ). HC: healthy control; CI: chronic insomnia.

**Fig. 3-3:**
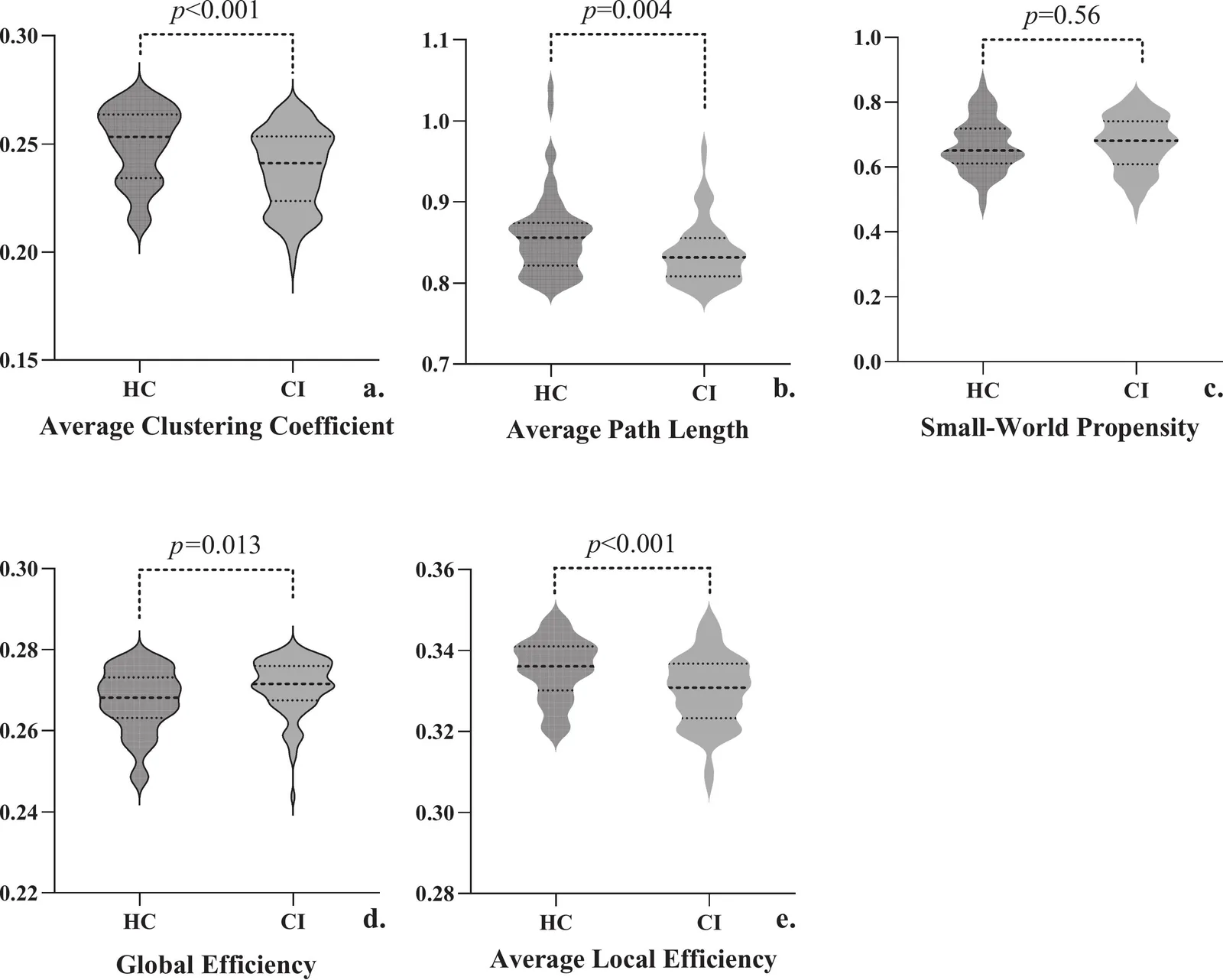
Group comparisons of global network metrics AUC values. HC: healthy controls; CI: chronic insomnia patients. Between-group differences were assessed using a GLM, and multiple comparisons were corrected using the Bonferroni method (corrected *p* < 0.01).

**Fig. 4:**
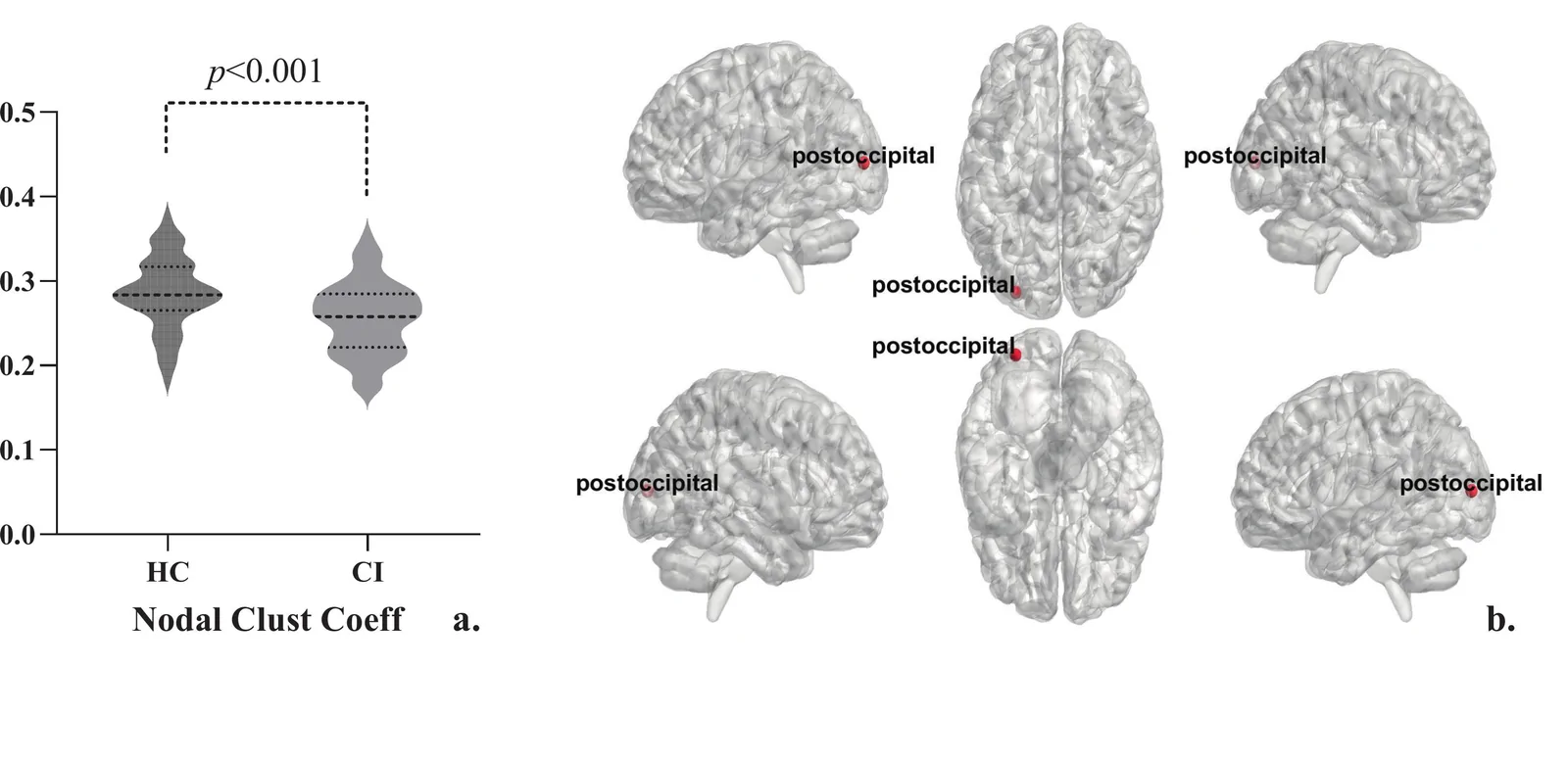
Group comparisons of nodal metric AUC values. HC: healthy controls; CI: chronic insomnia patients. Between-group differences were assessed using a GLM, and multiple comparisons were corrected with the Bonferroni method (corrected *p* < 3.13 × 10^−4^).

**Table 3:** Dosenbach atlas–based comparison of the topological properties of the CI and HC groups.

Measure	HC (*n* = 54)	CI (*n* = 97)	T (149)	P	Cohen's d	95% CI
Eg	0.267 ± 0.008	0.271 ± 0.007	−2.510	1.32E-02	−0.43	[−0.77, −0.09]
Eloc	0.335 ± 0.008	0.330 ± 0.009	3.594	4.46E-04	0.61	[0.27, 0.95]
Cp	0.250 ± 0.017	0.238 ± 0.018	3.887	1.54E-04	0.66	[0.32, 1.01]
Lp	0.859 ± 0.051	0.838 ± 0.039	2.897	4.35E-03	0.50	[0.16, 0.83]
Sigma	0.667 ± 0.083	0.672 ± 0.084	−0.582	5.61E-01	−0.10	[−0.43, 0.23]
Nodal Clust Coeff (posterior occipital)	0.285 ± 0.043	0.256 ± 0.043	3.947	1.23E-04	0.68	[0.33, 1.02]

Values are presented as mean ± SD. Between-group differences were assessed using a GLM with age, sex, years of education, and mean FD as covariates. Global metrics were corrected using the Bonferroni method (corrected threshold *p* < 0.01). Nodal metrics were corrected using the Bonferroni method (corrected threshold *p* < 3.13 × 10^−4^). CI, chronic insomnia; HC, healthy controls; Eg, global efficiency; Eloc, local efficiency; Cp, clustering coefficient; Lp, shortest path length; Sigma, small-worldness; Nodal Clust Coeff, nodal clustering coefficient.

### 6.1 Edge-wise connectivity results

NBS analysis identified one significant component. This component comprised 126 edges (approximately 63 unique edges after accounting for symmetry) involving approximately 50 nodes, predominantly located in the occipital cortex, sensorimotor regions, and parts of the frontoparietal network. The component-level FWE-corrected *p* value was *p* = 0.0034. All edges within this component showed reduced connectivity in the CI group relative to the HC group; no increased connectivity was observed. Edge-wise connectivity analysis (Fig. 5) revealed widespread decreases in functional connectivity in the CI group compared with the HC group. These reductions predominantly involved connections between parietal–posterior occipital, posterior insula–occipital, dorsal frontal cortex–temporal, dorsal frontal cortex–parietal, frontal–precentral gyrus, and precentral gyrus–occipital regions. Modular connectivity analysis further demonstrated that the CI group exhibited significantly reduced inter–modular connectivity between the sensorimotor network (SMN) and the occipital network (ON), and decreased intra–modular connectivity within the SMN itself (Fig. 6). Together, these findings suggest aberrant functional integration both across and within large–scale networks in CI.

**Fig. 5:**
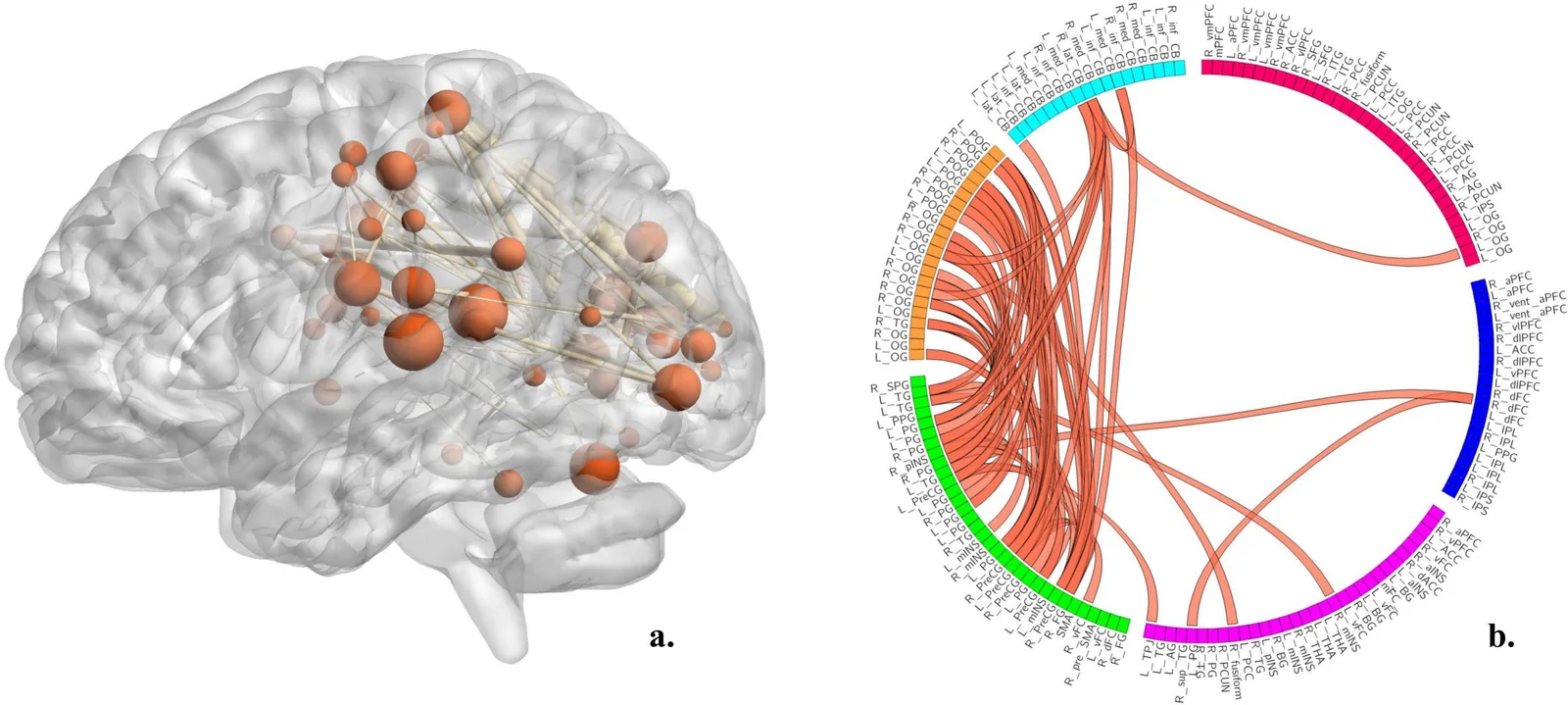
Brain map and Circos map of connectivity results. (a) Brain map. (b) Circos map. Statistical analyses were performed using a GLM, with NBS correction for multiple comparisons (*p* < 0.05).

**Fig. 6:**
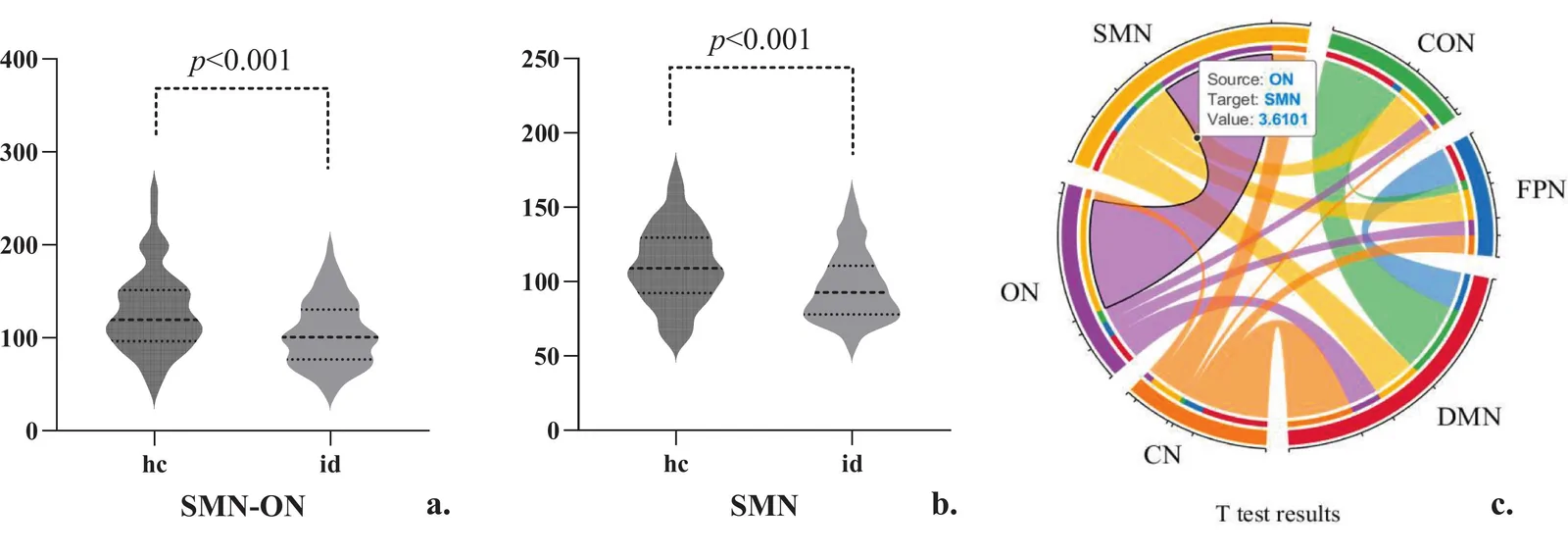
Inter-modular and intra-modular connectivity analyses. All statistical tests were conducted using GLM, with bonferroni corrected. (a) Inter-modular edge analysis (*p* < 3.33× 10^−3^). (b) Intra-modular edge analysis (*p* < 8.33× 10^−3^). SMN: sensorimotor network; ON: occipital network; (c) Chord diagram depicting the t-statistics derived from the inter-modular edge analysis.

## 7 Sensitivity and confirmatory analyses

To further examine the robustness of the main findings, we included SAS and SDS scores as covariates in all core between-group comparison models in a sensitivity analysis. The results showed that the effect directions and patterns of significance at the global, nodal, edge, and modular levels were consistent with the primary analyses. In addition, we used the Power-264 atlas as an independent alternative parcellation scheme and repeated the key graph-theoretical analyses. Global and nodal metrics showed consistent effect directions and patterns of significance across the two atlases, and the core findings at the edge and modular levels were also largely consistent with the primary results. These results indicate that the main findings of the present study remained robust after controlling for emotional symptoms and using an alternative brain atlas. Detailed results are provided in Supplementary Materials 1–3.

## 8 Discussion

In this study, by integrating graph-theoretical and edge-wise connectivity analyses, we systematically characterized the multi-level topological abnormalities in brain functional networks in patients with chronic insomnia. The principal findings are as follows: (1) at the global level, patients exhibited decreased average clustering coefficient, shortened shortest path length, and reduced local efficiency, alongside increased global efficiency, whereas small-world properties remained preserved, suggesting a shift toward a more randomized global topology with preserved small-world architecture and reduced local specialization; (2) at the nodal level, the left posterior occipital cortex showed a significantly reduced clustering coefficient, indicating impaired local integrative capacity; (3) at the edge level, widespread decreases in functional connectivity were observed across multiple pathways, predominantly involving parietal–posterior occipital, posterior insula–occipital, dorsal frontal cortex–temporal/parietal, frontal–precentral gyrus, and precentral gyrus–occipital connections; (4) at the modular level, both inter-modular connectivity between the SMN and ON and intra-modular connectivity within the SMN were significantly reduced, suggesting aberrant information exchange both across and within large-scale functional systems.

Collectively, these findings provide quantitative evidence for multi-level network dysfunction in chronic insomnia and highlight SMN–ON decoupling as a potential neural substrate and a candidate imaging biomarker for circuit-based interventions.

The sample size of the present study was sufficient to reliably detect between-group differences of moderate or larger effect size. The effect sizes for global and nodal metrics were predominantly medium-to-large, indicating robust findings. The effect size for small-worldness was small and non-significant, possibly reflecting limited statistical power to detect subtle effects. Future studies with larger samples are needed to validate these findings.

### 8.1 Global topological reorganization: co-existence of impaired local differentiation and compensatory long-range transmission

Normal brain functional networks exhibit small-world topological properties characterized by high clustering and short path length, enabling an efficient balance between local information integration and global information transfer (Bullmore and Sporns, 2009). Although previous studies have reported altered functional connectivity in CI patients, particularly involving the prefrontal cortex, limbic system, and default mode network (Jiang et al., 2016; Zhou et al., 2017), the pattern of whole-brain topological reorganization in CI remains to be fully elucidated. In this study, although multiple global topological metrics were significantly altered in patients with CI, small-world properties did not differ between groups, suggesting preservation of the fundamental network architecture, which is largely consistent with previous findings (Chen et al., 2024). Nevertheless, the observed decreases in average clustering coefficient and local efficiency, along with increased global efficiency, indicate a shift from a relatively localized information processing mode toward a more distributed global integration pattern. These topological alterations are consistent with those reported in a recent study examining the impact of insomnia on brain networks in depressed individuals (Chen et al., 2024). Typically, brain networks depend on strong local clustering for specialized processing and shorter path lengths for rapid cross-regional communication. The reduced local clustering observed here may reflect impaired stability of local functional modules in CI, whereas the elevated global efficiency may represent a compensatory mechanism that enhances long-range connectivity to sustain overall network function(Li et al., 2018; Menon, 2011). Nevertheless, excessive reliance on global integration may come at the cost of network segregation—a trade-off that reduces local processing efficiency and may underlie the impaired sleep regulation, emotional control, and cognition observed in CI.

Notably, the concurrent increase in global efficiency and decrease in local efficiency suggest that the network maintains global communication through augmented long-range transmission—a “compensatory reorganization”—potentially at the expense of local functional specialization. The shortened path length, which underpins the elevated global efficiency, further corroborates this compensatory interpretation. Despite preserved small-world properties, the network exhibits a distinct topological shift toward “enhanced transmission efficiency and attenuated local integration.” This reorganization may be associated with the reduced cognitive flexibility and altered sensory processing commonly reported in CI patients. Together, these findings support a model of co-existing local differentiation deficits and compensatory long-range transmission enhancements—a topological reconfiguration that may preserve global communication at the expense of specialized local processing.

### 8.2 Nodal-specific impairment: reduced hub function of the left posterior occipital cortex in local networks

Node-level analysis further revealed a significant reduction in the clustering coefficient of the left posterior occipital cortex in patients with chronic insomnia, may indicating impaired local network integration and a diminished hub role of this region within the functional connectome. The occipital cortex is a critical region involved in visual information processing and spatial attention regulation, serving as the primary entry point for visual input, with the parietal and temporal cortices extending the dorsal and ventral visual pathways, respectively. Previous studies have demonstrated that the visual cortex is not merely responsible for processing exogenous visual stimuli but is also closely involved in sleep-wake regulation, default mode network activity, and sensory information integration (Wang et al., 2022).

The observed alteration may be associated with the persistent hyperarousal state characteristic of chronic insomnia. On the one hand, sustained monitoring of environmental stimuli and heightened sensory vigilance may impose prolonged functional demands on the visual cortex, potentially disrupting local neuroplasticity and fine-grained functional regulation (Zhou et al., 2017). On the other hand, weakened connectivity between the occipital cortex and higher-order association regions, including the parietal and frontal cortices, as observed in our edge-based connectivity analysis, may further compromise its capacity for cross-regional information integration (Li et al., 2018). Previous studies have reported abnormal spontaneous activity and altered low-frequency oscillation amplitudes in the occipital cortex of patients with insomnia (Wang et al., 2025b), suggesting that occipital regions are involved in insomnia-related neural remodeling.

By applying graph-theoretical analysis, the present study extends these findings by demonstrating that CI-related occipital abnormalities are not confined to regional activity changes but involve impaired network-level integration, providing novel evidence for the role of the posterior occipital cortex in large-scale functional network reorganization in insomnia.

### 8.3 Edge and modular abnormalities: cross-system decoupling of the sensorimotor circuitry

Our evidence points to sensorimotor alterations as a key component of multisystem impairment, a finding that aligns with the hyperarousal model. Specifically, sustained vigilance may overdrive sensorimotor cortices, impairing their local integration and inter-network communication.

Edge-wise analysis revealed specific pathways of reduced functional connectivity in CI patients. Significant decreases were observed between the dorsolateral prefrontal cortex and temporoparietal regions, the frontal cortex and precentral gyrus, the posterior insula and occipital cortex, and the precentral gyrus and occipital cortex—largely consistent with previous findings (Li et al., 2026). The affected connections span prefrontal, parietal, temporal, insular, and motor systems, implicating both higher-order cognitive and sensory-motor domains. These findings extend the pathophysiological framework of CI beyond the limbic–prefrontal model to encompass sensorimotor and cross-modal systems. Although prevailing theories emphasize hyperarousal and emotion-regulatory dysfunction (Dressle and Riemann, 2023; Riemann et al., 2010), our evidence points to sensorimotor alterations as a key component of multisystem impairment, a finding that aligns with the hyperarousal model. Specifically, sustained vigilance may overdrive sensorimotor cortices, impairing their local integration and inter-network communication.

Modular analysis further revealed decreased inter-modular connectivity between SMN and ON, accompanied by reduced intra-modular connectivity within the SMN itself, which may constitute the neural substrate of daytime sensorimotor dysfunction in CI patients. This pattern of concurrent reductions in both intra-SMN and inter-SMN-ON connectivity suggests widespread functional alterations within the sensorimotor system in CI. The SMN subserves real-time somatosensory feedback and motor output, and its interaction with the visual network is essential for coordinating visually guided behaviors. The observed decoupling between these systems may compromise the efficiency of perception–action loops, clinically manifesting as daytime sleepiness, reduced motor coordination, and altered environmental perception. This sensorimotor-centered multi-network deficit not only extends conventional understanding of the neural mechanisms underlying insomnia, but also provides a novel network-level framework for interpreting clinically relevant daytime functional impairments, such as impaired sensorimotor performance and altered perception of environmental stimuli.

### 8.4 Mechanistic integration: a network perspective on hyperarousal and sensory gating deficits

Integrating the above findings, we propose a testable working hypothesis that the core network pathology in chronic insomnia may not be confined to canonical sleep–wake regulatory hubs, but rather manifests prominently as coupling failure within the sensorimotor–visual circuitry, accompanied by a global network shift toward randomization. Chronic hyperarousal imposes sustained high-demand loading on sensory processing systems, particularly the sensorimotor and occipital networks, potentially compelling the system to prioritize long-range communication over local integrative efficiency. This results in compromised fine-grained sensory filtering and integration, and is reflected in the observed reductions in local clustering and modular connectivity. Notably, these findings remained stable, and were even more pronounced, in the sensitivity analysis with SAS and SDS included as covariates and in the confirmatory analyses based on two independent parcellation schemes (Dosenbach-160 and Power-264).

This interpretation is consistent with the sensory gating theory of sleep: normal sleep depends on effective inhibition of external inputs, whereas patients with insomnia, because of impaired gating, fail to suppress irrelevant stimuli during sleep and cannot efficiently process relevant information during wakefulness. Notably, the observed reduction in posterior insula–occipital connectivity is particularly intriguing, as the posterior insula is a core hub for interoceptive and sensory awareness; its disconnection from visual regions may compromise cross-calibration between internal and external signals, thereby disrupting the neural differentiation between sleep–wake states. Collectively, these findings identify the SMN and ON as promising targets for future neuromodulatory interventions, thereby providing a theoretical foundation for developing circuit-specific strategies targeting the SMN–ON pathway.

### 8.5 Strengths and innovations

This study has several methodological and conceptual strengths. First, we established a multi-level analytical framework encompassing global, nodal, edge-wise, and modular metrics, enabling a systematic characterization of topological abnormalities in chronic insomnia. Second, to our knowledge, this is the first study to demonstrate concurrent reductions in both inter-modular connectivity between the SMN and ON and intra-modular connectivity within the SMN, thereby extending the classical frontal–limbic model of insomnia to include sensorimotor and occipital systems. Third, nodal analysis identified the left posterior occipital cortex as a locus of local integration deficits, highlighting a potential hub for future research on insomnia-related visual processing dysfunction. Finally, despite alterations in multiple topological metrics, small-worldness remained intact after rigorous multiple-comparison correction, suggesting that chronic insomnia involves selective, circuit-specific functional alterations rather than a global network breakdown. These findings remained stable in sensitivity and confirmatory analyses.

### 8.6 Limitations and future directions

Several limitations should be acknowledged. First, the cross-sectional case-control design precludes causal inferences. Whether the observed network alterations are causes, consequences, or compensatory correlates of chronic insomnia remains unclear and requires longitudinal and interventional studies to clarify. Second, although the sample size was moderate, subgroup analyses were not performed, and the relatively small sample size and imbalanced sex distribution in the HC group may have influenced between-group comparisons. In addition, the present study did not systematically record age at onset, previous treatment history, BMI, menopausal status, or long-term use of sleep-promoting and sedative-hypnotic medications. Future studies should employ design-stage matching or larger samples, incorporate multidimensional scales for mediation/moderation analyses, and systematically collect comprehensive clinical and medication histories to validate and extend these findings. Third, psychiatric disorders were not systematically excluded in the CI group, and subclinical emotional symptoms may have been present; although the supplementary sensitivity analyses were largely consistent, future studies should still systematically screen for psychiatric disorders at enrollment and use structured interviews to distinguish the independent effects of insomnia and emotional disorders.

Future directions include: (1) developing individualized predictive models using machine learning for patient stratification and treatment response prediction; (2) applying neuromodulation (e.g., rTMS or tDCS) targeting SMN or ON to verify therapeutic efficacy and network coupling; (3) integrating multimodal imaging (structural, functional, and metabolic) to explore white matter fiber pathways and neurotransmitter mechanisms; and (4) combining sleep microstructure features (e.g., spindles, slow waves) to bridge microscopic and macroscopic measures.

## 9 Conclusion

In summary, this study reveals that chronic insomnia is characterized by global topological reorganization (decreased local efficiency with increased global efficiency), reduced local hub function in the left posterior occipital cortex, and disrupted coupling between the SMN and ON and within the SMN. These findings provide novel evidence for multi-system network pathology underlying chronic insomnia and offer potential imaging targets for future intervention studies. Future studies with larger samples and longitudinal designs are warranted to validate the SMN-ON decoupling hypothesis and to examine its causal role in insomnia pathophysiology.

## CRediT authorship contribution statement

**Zuguang Wen:** Writing – review & editing, Writing – original draft, Visualization, Validation, Software, Resources, Methodology, Investigation, Formal analysis, Data curation, Conceptualization. **Yuefeng Kong:** Writing – review & editing, Resources, Formal analysis, Data curation. **Yaxuan Wen:** Resources, Data curation. **Hui Zhang:** Software. **Liling Long:** Writing – review & editing, Supervision, Resources, Project administration, Methodology, Funding acquisition, Conceptualization.

## Consent for publication

Not applicable.

## Ethics approval and consent to participate

The study was conducted in accordance with the *Declaration of Helsinki* and was approved by the Ethics Committee of the First Affiliated Hospital of Guangxi Medical University (Approval No. 2025-K0070). All individuals were informed of the study's purpose and significance, and they provided written informed consent.

## Ethics declaration

Written informed consent to take part in the study and to publish the article has been obtained from all participants or their legal representatives. The privacy rights of participants have been observed.

This study was performed in compliance with relevant laws, regulatory frameworks and guidelines where the research took place. This study was approved by the Ethics Committee of the First Affiliated Hospital of Guangxi Medical University. (Approval No. Approval No. 2025-K0070)

## Funding

This research received no external funding.

## Declaration of competing interest

The authors declare that they have no known competing financial interests or personal relationships that could have appeared to influence the work reported in this paper.

## Data Availability

Data will be made available on request.
